# Quality of palliative and end-of-life care: a qualitative study of experts’ recommendations to improve indicators in Quebec (Canada)

**DOI:** 10.1186/s12904-024-01474-8

**Published:** 2024-06-10

**Authors:** Emilie Allard, Sarah Dumaine, Martin Sasseville, Morgane Gabet, Arnaud Duhoux

**Affiliations:** 1 Faculty of Nursing, University of Montreal, PO Box 6128, Centre-ville Station, Montréal, QC H3C 3J7 Canada; 2 Centre de recherche Charles-Le Moyne (CRCLM), Campus de Longueuil - Université de Sherbrooke, 150 Place Charles LeMoyne - Bureau 200, Longueuil, QC J4K 0A8 Canada; 3 School of Public Health, University of Montreal, 7101 Av du Parc, Montréal, QC H3N 1X9 Canada

**Keywords:** PEoLC, Care access, Care equity, Quality improvement

## Abstract

**Background:**

In 2021, the National Institute of Public Health (INSPQ) (Quebec, Canada), published an update of the palliative and end-of-life care (PEoLC) indicators. Using these updated indicators, this qualitative study aimed to explore the point of view of PEoLC experts on how to improve access and quality of care as well as policies surrounding end-of-life care.

**Methods:**

Semi-directed interviews were conducted with palliative care and policy experts, who were asked to share their interpretations on the updated indicators and their recommendations to improve PEoLC. A thematic analysis method was used.

**Results:**

The results highlight two categories of interpretations and recommendations pertaining to: (1) data and indicators and (2) clinical and organizational practice. Participants highlight the lack of reliability and quality of the data and indicators used by political and clinical stakeholders in evaluating PEoLC. To improve data and indicators, they recommend: improving the rigour and quality of collected data, assessing death percentages in all healthcare settings, promoting research on quality of care, comparing data to EOL care directives, assessing use of services in EOL, and creating an observatory on PEoLC. Participants also identified barriers and disparities in accessing PEoLC as well as inconsistency in quality of care. To improve PEoLC, they recommend: early identification of palliative care patients, improving training for all healthcare professionals, optimizing professional practice, integrating interdisciplinary teams, and developing awareness on access disparities.

**Conclusions:**

Results show that PEoLC is an important aspect of public health. Recommendations issued are relevant to improve PEoLC in and outside Quebec.

**Supplementary Information:**

The online version contains supplementary material available at 10.1186/s12904-024-01474-8.

## Background

The World Health Organization [1] defines palliative care as “an approach that improves the quality of life of patients (adults and children) and their families who are facing problems associated with life-threatening illness. It prevents and relieves suffering through the early identification, correct assessment and treatment of pain and other problems, whether physical, psychosocial or spiritual”. Even if palliative care should be integrated earlier in the illness trajectory, in this study the term Palliative and End-of-Life Care (PEoLC) represent the palliative care offered in the advanced stages of illness and at the end-of-life. In 2015, the Quebec government (Canada) passed The Act Respecting End-of-Life Care, which recognized the importance, for all Quebecers, to have access to good quality palliative and end-of-life care for the entire territory. A commission, composed of 11 government-appointed experts, was mandated to examine the status of palliative care in the province. In their first annual report, the commission concluded that a lack of reliable data and indicators made it difficult to get an accurate depiction of the current state of palliative care in Quebec [2]. In 2006, the National Institute of Public Health (INSPQ) published a report assessing 10 PEoLC indicators, using administrative data from 1997 to 2001 [3], since then these indicators weren’t properly assessed and monitored.

A two-part research project carried out jointly with the INSPQ was undertaken to respond to the lack of data and to suggest ways to improve PEoLC. Using administrative data from 2002 to 2016, the first part of the study (quantitative) led to the publication of a report assessing 8 PEoLC indicators [4] and an article recently published [5] While indicators based on administrative data are useful to assess PEoLC on a populational-level, they are best used when combined with other methods of measurement, such as qualitative data. Consulting key actors and experts is central to assess the validity and usefulness of PEoLC indicators and can lead to an increase in their acceptance and perceived validity among the palliative care community [6]. Therefore, this article presents the results of the second part of the study (qualitative) aimed at interpreting and issuing recommendations on the PEoLC indicators.

The indicators assessed from 2002 to 2016 (Appendix 1) relate to the place of death, emergency room (ER) use, hospitalization, and care received at the end of life [4, 6]. The indicators targeted patients of varied biological sex, age, illness trajectories and place of residence, who would likely have benefited from palliative care prior to death. The three illness trajectories referred to: terminal illness (Trajectory I), organ failure (Trajectory II) and frailty (Trajectory III). From 2002 to 2016, the number of patients who would likely have benefited from palliative care prior to death increased (+ 15.3%). Home deaths increased non-significantly (+ 1.4%), accounting for less than 10% of total deaths in 2016. Deaths in residential and long-term care facilities increased (+ 4.51%), representing a quarter of the total deaths in 2016. These numbers were associated with an increase in Trajectory III patients, who died predominantly in these settings. Deaths in palliative care homes rose threefold (3–9.8%), while remaining nearly exclusive to Trajectory I patients. General and specialized care hospitals continued to be the leading place of death, especially for Trajectory II patients (67%), but deaths there decreased (65.9–53.1%). In acute care beds with no palliative care, deaths dipped (39.7–21.4%) while Trajectory II patients remained overrepresented. Trajectory II and III patients were less likely to receive palliative care in hospitalizations leading up to death. Moreover, 42.2% of patients who would likely have benefited from palliative care prior to death visited the ER at least once in their last two weeks of life. This was higher in Trajectory II patients (52.6%) and lower in Trajectory III patients (20.9%). Furthermore, 14.4% of patients visited the ER on the day of their death, 8.4% of them died or had their death declared in the ER, and resuscitation acts were attempted on 25% of them. Interregional variations were observed regarding ER use at the end of life and in palliative care received during hospitalizations leading up to death.

The aim of this qualitative study was to better understand how the proposed indicators reflect the reality of PEoLC in Quebec while issuing recommendations to improve data and care at the end of life.

## Methods

### Design

An exploratory design was used to engage with the palliative care community [7], such as healthcare professionals, service planners and policy actors, to ensure better understanding and use of administrative data in PEoLC.

### Sampling and expert identification

Sampling was informed by a snowballing approach, where experts were identified by members of the research team and other experts based on their professional experience with PEoLC or policy making in Quebec (Canada). To gain a sense of regional variations in data, experts from different regions were invited. Since PEoLC are interdisciplinary in nature, attention has been paid to the disciplinary background and workplace of the experts approached for the interviews. This sampling strategy was pursued until data saturation, i.e. redundancies in the themes of the analysis.

### Data collection

The first author [EA] conducted semi-directed interviews with nine participants. Prior to the interview, an Excel spreadsheet summarizing the updated PEoLC indicators assessed from 2002 to 2016 was sent to all the participants. Each interview lasted approximately 60 min and was audio recorded to ensure an integral transcription. These interviews were conducted in person or by phone, in a place and time of choice of the participants. During the interviews, participants were first asked to share their interpretation of the data and indicators as well as compare this information to what they observe in their field or practice. They were also asked to offer recommendations to improve PEoLC in Quebec (interview guide available as Supplementary File 1).

### Data analysis

Each interview was coded independently by a student and the first author [EA] using thematic analysis [8]. Each unit of analysis (i.e. sentences on the same subject) was coded using a word or group of words related to the comment made by the participant. The codes shaped a thematic tree that was used to code the next interviews. The codes from each interview were compared to identify similarities and differences. Common codes were gathered in more comprehensive themes and are presented in this article. Excel was used as a qualitative data analysis tool [9].

## Results

A total of nine interviews with PEoLC experts from different disciplinary fields including medicine, nursing, social work, pharmacy, and policymaking. They reside in four different regions of Quebec, regional and urban settings, helping us reach a broader description of the PEoLC reality in the province. They worked in varied settings including university, hospital, long-term care, hospice and home care. Most of them also had experience in research or teaching, therefore were comfortable with the research process and understand how to read large data set and indicators.

The analysis of the interviews leads to themes pertaining to participants’ interpretations (I) regarding the updated PEoLC indicators, as well as their recommendations (R) to improve accessibility and quality of PEoLC in Quebec. These interpretations and recommendations were regrouped into two main themes, regarding: (1) data and indicators and (2) clinical and organizational practice. The results are summarized in Table 1.

**Table 1:** Participants’ interpretations and recommendations

Participants’ interpretations (I) and recommendations (*R*)		
**Regarding data and** **indicators**	I1	Lack of reliability of the collected data
	R1	Improve the rigour and quality of the collected data
	R2	Assess death percentages in all healthcare settings
	I2	Lack of data and indicators assessing the quality of care
	R3	Promote research on quality of care
	R4	Evaluate care intensity and continuity
	R5	Compare data to EOL care directives
	R6	Assess the use of ER in EOL
	R7	Create an observatory on PEoLC
**Regarding clinical and organizational practice**	I3	Existence of multiple barriers to palliative and EOL access • Three-month or less prognostic • Late referrals due to scarcity of beds • Lack of public awareness • Healthcare professional reluctance
	R8	Early identification of palliative care patients
	R9	Better training on PEoLC
	I4	Inconsistent and substandard quality of care
	R10	Better integration of the whole interdisciplinary team
	R11	Optimization of professional practice
	I5	Disparities in care
	R12	Develop awareness on disparities and vulnerabilities in PEoLC

### Data and indicators

The first theme regards the quality and reliability of the PEoLC data and indicators as well as the lack of data regarding quality of care.

Participants addressed the lack of reliability in data collection from administrative databases (I1). They argued that data collection and entry methods differ by profession, care setting, and region, making it difficult to draw reliable conclusions from an administrative data set alone. For example, one participant brought up that ER deaths may be declared as home deaths if the death occurred less than 24 h after ER admission, this practice was not the same in other regions. Another participant described that many palliative care consultations are entered in databases but never take place because patients die while waiting for the consultation. Such practices could have a distorting effect on data and make it inaccurate for research or monitoring purposes.

Participants thus recommended improving the rigour and quality of PEoLC data (R1). Knowing that the Quebec government closely tracks some indicators (e.g. home death), participants expressed that data collection must be systematic, transparent, and independent of management and accountability agreements. They also stressed the need for the government to assess death percentages in all healthcare settings, not only in the home care context (R2). Even if dying at home is a common idea of what constitutes a “good death”, there are many reasons why all deaths do not or cannot occur at home. Therefore, the percentage of home deaths alone is not an indicator of palliative care access or quality.

All participants criticized the lack of data and indicators measuring the quality of care provided (I2), without which it is impossible to truly assess PEoLC in Quebec. Participants expressed the need for data assessing the amount of care received, and its efficacy in addressing patients’ physical, psychological, and spiritual needs. Moreover, they disagreed with solely using the number of home deaths to assess at-home palliative care. They argued that patient preferences regarding the place of death, caregivers’ needs, as well as the amount and quality of at-home palliative care, needed to be assessed alongside this indicator.

To encourage the assessment of PEoLC quality, participants recommended more research on quality of care (R3), such as retrospective and prospective studies on medical records, and qualitative research exploring patient and family perspectives on quality of care. Another recommendation was to evaluate care intensity and continuity of care (R4): beyond the number of services, the monitored indicators should focus on the appropriateness, intensification, and continuity of services offered at the end of life. Cross-referencing PEoLC data to medical directives (R5) was also recommended, as care can be considered of good quality when it meets the end-of-life wishes laid out in patients’ medical directives. Additionally, the number of and reasons for ER visits at the end of life should be added to the list of government-monitored indicators in Quebec (R6) as differentiating preventable from non-preventable visits would help identify weaknesses in service quality and continuity, particularly at-home PEoLC.

Finally, to encourage the development of more reliable and thorough PEoLC data and indicators, participants recommended that the government create a national end-of-life observatory (R7), an independent body whose mission is to support data-based decision making. They argued that an observatory would ensure continuous access to and transparency of data as the palliative care context evolves and would shed light on data weaknesses, stimulating more data collection efforts.

### Clinical and organizational practice

The second theme regards the issues inherent to clinical and organizational practice that reduces access, quality, and equity in PEoLC.

Comparing the data in the three illness trajectories (I, II and III), participants named barriers in accessing PEoLC in care settings. Among others, they mentioned that early integrated palliative care is misunderstood, which mainly affect Trajectory II and III patients, owing to their uncertain prognosis and slower evolution towards death. Another barrier to the early integrated care mentioned by the participants is the three-month-or-less requirement for accessing many palliative care services, such as at-home palliative care, hospital palliative care units, and palliative care homes (hospices). Some participants also discussed how palliative care units, functioning with limited resources and scarce beds, often put pressure on clinicians for late referrals to limit the length of stay of each patient. Meaning that patients are transferred later and later in their illness trajectories, reducing the ability to adequately support and relive suffering. Resulting in the reinforcement of the belief that palliative care is offered only at the end of life. Participants also believed that access to care is hindered by lack of patient awareness of what constitutes palliative care. According to them, patients are often unaware of documentation such as advance care planning, and little effort is made by healthcare professionals to discuss this documentation. Participants also argued that healthcare professionals, such as physicians and nurses, are often reluctant to discuss PEoLC with patients and their families. They may struggle to determine the appropriate time to stop curative interventions, especially when a patient’s condition is chronic and not traditionally associated with palliative care, such as in Trajectory II patients. Participants believed that such barriers could explain the data related to the decreased amount of time spent in palliative care services prior to death (7 days in 2002 to 3 days in 2016), as well as Trajectory II and III patients’ limited access to PEoLC services compared to Trajectory I patients.

To address these barriers, participants notably recommended the early identification of palliative care patients (R8). Identification tools should be developed and implemented to improve early identification, regardless of illness trajectory. Participants also argued that healthcare professionals should receive better training regarding palliative and end-of-life care (R9). They argued that with the aging population and the increase of chronic diseases, palliative care teams can’t be the sole caring for the patients living with serious illnesses. Participants suggested that mandatory palliative and end-of-life care classes should be added to every healthcare curriculum to ensure a minimum skillset. They also suggested that overall public awareness about palliative care should be raised to improve shared responsibilities on access to these services. Moreover, they recommend setting up an interdisciplinary team in every care setting (R10), with high-level palliative care competencies, to support the other care teams.

Once accessed, care quality is often inconsistent and even substandard, the participants argued. Poor quality was mainly described in at-home palliative care, which participants attributed to lack of funding and human resources in community care settings. Participants claimed that limited resources forced at-home palliative care professionals to spread out and shorten their visits, leading to a more biomedical approach that leaves out the psychological, spiritual, and social dimensions of palliative care. Some participants also claimed that nurses, involved in at-home palliative care, couldn’t exert their full scope of practice and lacked professional autonomy when intervening with patients. It was believed that the substandard quality of at-home palliative care could help explain the high use of the ER at the end of life, as patients are referred or turn to the ER for acute health issues that at-home palliative care fails to address. Concerns were expressed about “palliative” patients being placed in medical-surgical units following a recent reform in Quebec healthcare. Participants argued that these units were inappropriate for the dispensation of PEoLC among other things because of their high nurse–patient ratio, which don’t allow for the consideration of patients’ psychosocial and spiritual needs and because of the lack of training of the healthcare professionals in palliative and end-of-life care. Participants believed that Trajectory I patients encountered fewer quality issues in palliative care, as they had access to more resources and received better follow-up, given that their illness trajectory is more certain and that oncologists tend to be better trained in palliative care.

To address quality issues, participants recommended optimizing professional practice (R11), especially for registered nurses in at-home palliative care. Participants stressed the importance of developing professional practices to their full potential, along with tools and resources (e.g. collective prescriptions) for more effective management of PEoLC situations. Participants also recommended better integration of the whole interdisciplinary team (R10) as the management and support of palliative care patients and their families requires a complete, fully trained interdisciplinary team that includes a social worker, a spiritual care worker, and more. Once again, the participants called for better training in palliative and end-of-life care for all healthcare professionals (R9).

Looking at the data by regions, participants identified disparities in access to PEoLC. They believed care was more developed in certain regions of Quebec. Some participants wondered about whether the high percentage of home deaths in northern Quebec could be attributed to cultural characteristics of its Indigenous communities or to insufficient alternative services. A desire was expressed to obtain and track data on vulnerable populations, such as cultural minorities and the homeless community. Inequities were felt to pertain to cultural and regional differences, care settings, and disease trajectories. Raising awareness of disparities and vulnerabilities in PEoLC (R12) was called for, stating that the government must consider these factors if it is to improve services.

## Discussion

Drawing on participants’ interpretations of the INSPQ data and indicators, this study aimed at issuing recommendations to improve the quality and accessibility of PEoLC in Quebec. The findings pertain to two aspects: (1) data and indicators, and (2) clinical and organizational practice.

PEoLC are a public health concern [10]. To implement and improve PEoLC it is important to have access to good and reliable data and indicators. However, regarding PEoLC data and indicators, participants addressed two main areas of concern: the lack of reliability of the collected data, and the lack of data assessing quality of care. As shown in recent findings [4, 6], most indicators used in Quebec to measure PEoLC are based on administrative data. Administrative data are being used because they are considered effective and inexpensive [11, 12]. Their routine collection also allows for constant monitoring and historical, regional, and international comparisons [7, 11]. The downsides of administrative data mentioned by the participants resonate with the literature. Lack of standardization in data collection and coding may affect its reliability and comparability, and ultimately undermine conclusions drawn from it [7]. While having administrative data has its limits, improving its reliability is possible. Davies et al. [7] suggest that data holding bodies should publish their data and increase collaborative relationships with data experts and the wider palliative care community to improve transparency and the creation of more innovative projects based on data. Similarly, participants in our study suggested that a nonpartisan and independent organization, namely an end-of-life observatory, should be the body to assess and ensure data governance.

The participants of our study also recommended monitoring the percentage of deaths in all healthcare settings, as opposed to the government solely monitoring home deaths. While home death has been identified as a valid and accepted indicator of appropriate end-of-life care, this is also contested since preferences regarding the place of death are deemed highly personal and evolutive in the dying trajectory [13]. The literature shows that studying the place of death makes it possible to identify care settings that play the most important roles in delivering PEoLC, which can then guide resource allocation for improving palliative care [14, 15]. Monitor all places of death could help assess the impact of policies and programs and could allow for a comparison of desired vs. actual place of death, as well as interregional and international comparisons [14, 15].

The issue related to the lack of data on the quality of care was mentioned by the participants and also looked at in the literature on the use of administrative data for monitoring, as it fails to address important aspects of palliative care, such as the management of physical, psychosocial, and spiritual needs, and the communication of advanced directives [12]. The literature shows that, to better assess PEoLC, administrative data should be used in conjunction with other methods to help contextualize data and indicators, such as patient-reported outcome measures (PROMs), quality indicators or qualitative data [11, 12, 16]. Listening to the voice of those who provide care and those who received care could help shed light on issues related to PEoLC, issues not necessarily evident in administrative data. Administrative data could also be cross-referenced with documented end-of-life care directives. For example, the percentage of deaths occurring in patients’ preferred location is a national palliative care indicator in the Netherlands [17]. Leading countries in palliative care, namely the Netherlands, Belgium, Sweden, and the US, also consider documented medical directives (e.g. treatment preferences and care objectives) when assessing PEoLC on a national level [17], showing that this comparison is both feasible and relevant. Another recommendation issued by the participants is to include ER use in end-of-life among the indicators monitored by the government within management and accountability agreements, as a high ER use has been identified as an indicator of poor quality PEoLC [5, 11, 13, 16].

The participants interviewed addressed three main concerns regarding clinical and organizational practice: the multiple barriers to PEoLC access, inconsistent and substandard quality of care, and disparities in care. It is often difficult to pinpoint the optimal time to introduce palliative care and the healthcare professionals who should deliver it. However, early identification of patients likely to benefit from palliative care is linked to better quality of life and to better life expectancy [18, 19]. However, early identification measures can be subjective and challenging. The use of accessible, validated, and evidence-based tools, such as the Supportive and Palliative Care Indicators Tool (SPICT), may facilitate and standardize early identification of patients likely to benefit from palliative care, all trajectories and care settings alike [20]. Educating professionals on the role and benefits of early palliative care could increase patient referrals by reducing barriers associated with its traditional view as EOL care [19]. Better training is also required to tackle disparities in access to palliative care for patients suffering from chronic conditions (Trajectory II).

Aside from better training, the participants also recommended that professional practice be optimized and the whole interdisciplinary team be better integrated to ensure quality of care. Involving a multidisciplinary team in cancer and chronic disease allows for better symptom management, better coordination of care, reduced caregiver burden, improved cost effectiveness, fewer admissions, and more patients dying at home [21]. In an integrated palliative care model, general healthcare professionals, trained in palliative care, should tend to many of the patients’ needs and consult specialized palliative care teams for more complex and refractory problems [22].

Regarding the recommendation to raise awareness of disparities and vulnerabilities in PEoLC, the literature shows that cultural differences and communication barriers may hinder access to palliative care in culturally diverse patients [23, 24]. Developing awareness of differences in palliative care delivery between healthcare settings may also be relevant in addressing issues of access and quality of care. Participants highlighted inconsistent and substandard quality in at-home palliative care in Quebec, but this has also been found in the Canadian province of Ontario [25]. Awareness regarding differences in palliative care delivery between trajectories is also relevant, as Trajectory II and III patients face more barriers to palliative care access than Trajectory I patients [19, 26, 27]. Even if access to quality PEoLC is recognized as a human right, structurally vulnerable people (e.g. first nations, homeless, migrant) don’t have access to these services, meaning that public policy and resource allocations are not equitable in our society [27, 28].

## Conclusions

Most recommendations issued by the participants are feasible and relevant to improve PEoLC monitoring, research, accessibility, and quality in Quebec. These recommendations are also relevant for regional and national use outside Quebec and can be used by policymakers worldwide. As mentioned by the Lancet Commission on the value of death, “death is part of life” and in that sense death is part of a public health movement bonding policy making to every citizen [27]. Therefore, we need reliable data and indicators to shed lights on the gaps surrounding PEoLC in our healthcare system and to inform and improve care for everyone.

### Electronic supplementary material

Below is the link to the electronic supplementary material.

Supplementary Material 1

## Data Availability

Not applicable.

## **Appendix 1** PEoLC indicators

**Table:**

Indicators	Wished direction	Observed direction from 2002 to 2016
1. Percentage of home deaths that would have been likely to benefit from palliative care	↑	↔
2. Percentage of deaths in acute beds (with no visit to palliative care services) in patients that would have been likely to benefit from palliative care	↓	↓
3. Percentage of patients that would have been likely to benefit from palliative care with one visit or consultation in palliative care prior to death	↑	↑
4. Percentage of patients that would have been likely to benefit from palliative care having been hospitalized 14 days or more in last month of life	↓	↔
5. Percentage of patients that would have been likely to benefit from palliative care having been hospitalized 2 times or more in the last month of life	↓	↔
6. Percentage of patients that would have been likely to benefit from palliative care having visited the ER at least once in the last 14 days of life	↓	↔
7. Percentage of patients that would have been likely to benefit from palliative care having visited the ER the day of their death or having their death declared in the ER	↓	↔
8. Percentage of patients that would have been likely to benefit from palliative care having visited the ICU at least once in the last month of life	↓	↔
↓ = decrease; ↑ = increase; ↔ = no significant changes		
