# Supplementary material for: Quality of palliative and end-of-life care: a qualitative study of experts’ recommendations to improve indicators in Quebec (Canada)
Source: BMC Palliat Care. 2024 Jun 10;23:146. doi: 10.1186/s12904-024-01474-8 (PMC11163802; doi:10.1186/s12904-024-01474-8)
Supplement: Supplementary file 1 — Supplementary Material 1 [file 12904_2024_1474_MOESM1_ESM.docx]

**Supplementary file 1. Interview guide.**

Introduction

First, we would like to thank you for agreeing to participate in this project, whose ultimate goal is to contribute to improving the quality and accessibility of palliative and end-of-life care (PEoLC) in Quebec. The interview will last approximately 60 minutes. The purpose of the interview is to gather your interpretation, as an expert in palliative and end-of-life care, of the indicators identified in the first phase of the research project, as well as your recommendations regarding the evolution of palliative end-of-life care.

*Before you begin*

1. In order for me to get to know you, I would like you to briefly introduce yourself (name, discipline, experience in palliative and end-of-life care, practice setting, etc.).

*General impression*

2. When you look at the portrait of the indicators, what is your first impression?

3. How does this picture relate to your personal and professional experience with PEoLC in Quebec?

4. Have you noticed any changes in the picture of PEoLC since the law on end-of-life care came into effect in December 2015?

*Interpreting the indicators*

Let's now look at each of the indicators individually. I would like you to share your understanding of these indicators with me.

5. What is your interpretation of them? How do you analyse each of these indicators in relation to your personal and professional experience of PEoLC?

- Place of death

- Resources

- Emergency services at end of life

- Interventional procedures

- Hospital stays

- Care during hospitalization leading to death

*Recommendations*

6. In light of these indicators, what recommendations would you make to improve the quality and accessibility of PEoLC in Quebec?

7. In your opinion, what are the factors that facilitate and constrain the quality and accessibility of PEoLC in Quebec?

*Conclusion*

Would you have anything else to add?
